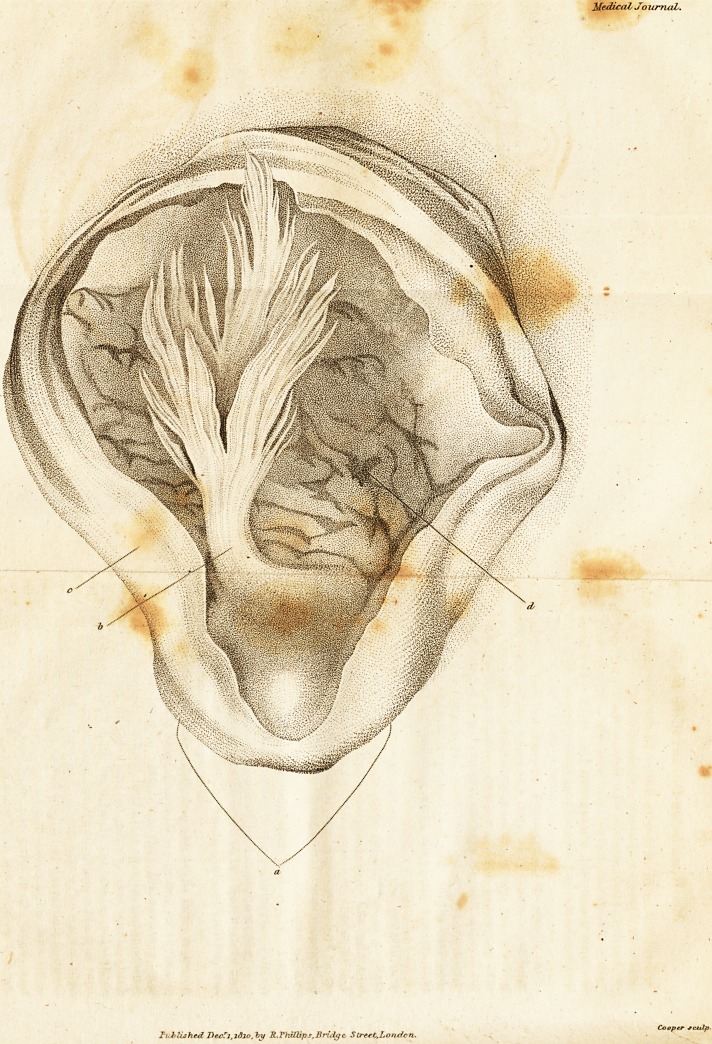# Observations on Diseases of the Brain, with Cases and Dissections

**Published:** 1810-07

**Authors:** J. Howship

**Affiliations:** Surgeon


### Observations on Diseases of the Brain, with Cases and Dissections.

By Mr. J. Howship, Surgeon.

*A Case in which Mercurial Excitement, instituted for the Removal of Venereal Complaints, was translated from the salivary Glands to the Brain, producing violent Attacks of Convulsion.*

( In Continuation. )

J. COYLE, aged 30, private in the 82d regiment of foot, was admitted into hospital, December 1, 1808, with inflammation and œdematous tumour of the prepuce, paraphymosis, and bubo. These complaints were severely painful; the tumefaction of the prepuce was such, as to produce constant uneasiness from its creating a sense of stricture and tightness around the penis, just behind the corona glandis. This state continued much in the same degree for about a week, when the effusion that had taken place in the cellular membrane of the prepuce suddenly increased to an alarming extent. Upon this account the prepuce was divided; a director was introduced within the edge of the preputium, upon the superior part of the penis. Upon the director a strait sharp pointed bistoury being passed up, the point was pushed through the integuments, and the instrument made to cut its way out The divided edges of the wound immediately separated to the extent of an inch, and and the livid colour which had for some time rendered the safety of the glans penis doubtful, very soon disappeared.

The bubo had been fomented and poulticed for some days, when an opening was made, and a considerable quantity of matter let out.

Between the 1st and 13th of the month he had rubbed in four ounces of the strong mercurial ointment; on the 14th the frictions were suspended; not that the appearances of the disease were in the least degree altered for the better, but on account of the very severe state of excitement that had taken place in the salivary system. By this time the wound upon the prepuce had formed a spreading, painful, and highly inflamed chancrous sore. The opening in the bubo had increased to a very extensive ulcer, retaining its venereal characters.

From the 14th to the 24th, little was done; astringent gargles were frequently used. On the 25th, the swelling of the face and excessive soreness of the mouth were so nearly removed, that he was again ordered to rub in one drachm of the mercurial ointment every night. This treatment was continued up to the 17th of January following, when it became a question whether it would be right to persevere in the frictions, although the appearance of the ulcerations was by no means healthy.

The bubo had produced a very extensive ragged ulceration, the edges of the skin were livid, indurated, and disposed to turn inward upon the sore, while the surface of the ulcer exhibited a mass of forward granulation, without the least disposition to heal. The ulceration behind the glans was still increasing in extent, and at times was very painful.

With this state of the disease, the energies of the system were so much reduced, that the man was scarcely able to rise from his bed. The pulse also was so very weak and low, that it was with difficulty it could be perceived at the wrist.

He was now, therefore, ordered the preparations of bark, the further use of mercury being postponed. Under this treatment the strength and constitutional powers improved daily, and the tonic plan was continued till the seventh of February, when he appeared to be very materially stronger and better in health, although still weakly. The Inspector of hospitals being at this time on his way through the district, he visited the hospital. A consultation was held upon this man's case, the result of which was, that he was again ordered to rub in one drachm of

 the the mercurial ointment every night. The ulcers were still in the most unhealthy state; the irritation in the salivary glands was now so far allayed, that he was enabled to continue the frictions without intermission, from the 7th to the 24th of February. The mouth at this time became again very sore, with an attendant state of high excitement in the system, and the most excessive irritability. The frictions were therefore alternated with opiate and tonic medicines, according to the daily variations in the general health.

On the 28th of February his pulse and strength were reduced to so hazardous a degree of exhaustion, that it was deemed necessary to relinquish the use of mercury altogether, notwithstanding the excessive pain and constant distress he suffered from the still spreading ulcerations.

Upon the 2d of March, he was ordered the compound decoction of sarsaparilla, to be taken frequently as the ordinary drink. This agreed very well, and the following day he said he found himself much better; he felt stronger, had rested better at night, and had more appetite than before. The decoction was continued, and he drank near two pints daily.

March the 6th. He had passed a restless night, from a very sharp, acute, and shooting pain in the ulcer upon the prepuce; upon examination there was no material change in the appearance of the sore. Twenty drops of laudanum were ordered to be given at bed time.

March 7th. He was up, and thought himself better, but towards the evening he lay down, and found himself poorly.

March 8. From having taken cold the preceding day, he had been in the evening attacked suddenly by a most violent pain in the head, during which he rose up from his bed, and sat himself by the fire in his shirt; but of this circumstance he never had the smallest recollection. He had passed a tedious night, and this morning complained of frequent darting pains shooting through his head; although much better than in the night. He had also, frequently, a severe pain extending down his arms to his hands; the pulse was soft and small, not beating more frequently than 80 in the minute.

March 9. The affection of the mouth almost subsided; in other respects much the same. Pain in the head sometimes severe. He took this evening fifteen grains of the compound powder of ipecacuanha.

March 10. He was rather better. The evening powder was repeated.

March

March 11. To day he said that the rolling noise and pain in the head were much relieved, and that he found his general health greatly improved; his appetite was good, and his bowels regular, not disposed to costiveness. From this date to the 21st of the month he seemed to mend, taking every evening ten grains of the compound powder of ipecacuanha.

March 22. The pulse was so weak, soft, and small, that it could scarcely be felt. The diet drink certainly gave tone to the appetite, which was now very good, and added to the general strength; the surface of the ulcer above the glans penis had in the centre assumed a livid, blackish colour, and in a few days threw off a sloughy film. After the separation of the slough, opiates were discontinued; he was then ordered two grains of the extract of bark twice a day, and this tonic appeared to answer very well. He gained strength.

While the penis was exceedingly painful, a few days previous to the formation of the little slough, a poultice of fresh grated carrot was applied one evening, but it aggravated the pain so greatly that the night passed in a state of delirium. The day following a warm poultice of bread and milk was substituted, and this gave immediate relief, and with this he the next night slept soundly without an opiate.

March 28. The health improving slowly; pulse 100; gaining some little substance. The diet drink continued.

March 30. Had so violent a pain in the head that the bark was given up, and a blister applied to the nape of the neck.

April 2. As the pain in the head still continued to return by fits with great severity, and as these complaints had, in this man, been frequently relieved by opiates, ten grains of the compound powder of ipecacuanha were directed to be given in the evening.

April 4. This evening, about seven o'clock, he was suddenly attacked with a most excruciating pain in his head, which very quickly deprived him of his reason, and he recollected nothing of what followed. He was laid upon his bed, and soon afterward fell into a fit. A general state of spasm and convulsive action of the muscular parts of the body and limbs, by which the organs of respiration were especially affected. The chest appeared to be fixed, and the jaws locked closely together. The violence of the attack in the space of half a minute began to abate, and the breath returned.

Of these attacks he had three within the hour. During the the intervals, he was, at times sensible, and complained much of a rolling heavy pain passing through his head. His general state was that of low muttering delirium. Upon examination, the pupil was found to retain its sensibility and power of contraction on the application of light. The pulse was exceedingly soft and small; it was quickened to 100 in the minute.

This poor man was already reduced to so low a state, that although his life evidently depended upon something that was to be done immediately for his relief, yet bleeding was quite out of question. Neither did the producing evacuation from the bowels seem to afford much ground for hope. If mild, they would have proved useless; if violently drastic, it was to be expected that they might very probably prove fatal by draining away the little energy that remained in the body.

It was very probable that one cause of the present mischief, if not the principal, was the suppression of the late profuse discharge from the salivary glands. The affection of the mouth had been very severe, and had almost totally subsided at the time the pain in the head was first complained of. From these circumstances it was determined to bring on, as quickly as possible, the same state of excitement in the salivary system, that had lately existed. A large blister was laid between the shoulders. Fifteen grains of the compound powder of ipecacuanha were given; and mercurial ointment was directed to be immediately and assiduously rubbed in about the trunk and extremities, until three ounces were consumed.

April 5. He had suffered during the night from repeated and very severe attacks of convulsion; in one of these the breath was so long in returning, that his recovery was scarcely expected. About half after two in the morning, he had the last fit, previous to which, the whole three ounces of ointment had been fairly rubbed in. At three he seemed to be recovering his recollection. He was rational, and said that he was "much better." As early as four in the morning he called for his handkerchief, and said "my mouth runs already." By ten o'clock he was quite sensible, and always free from the pain in the head, and the noise in the ears. The pulse was low and soft, beating 88. The blister between the shoulders had been in the course of the night torn off. Another was therefore applied to the chest.

As the full effect of the remedy seemed still to be somewhat doubtful, another ounce of the mercurial ointment was

*Published Decr. 1, 1810, by R. Phillips, Bridge Street, London.*

*Cooper sculp.*

was rubbed in by twelve to day. In the afternoon his mind was wandering, and clear at intervals.

April 6. He had slept well during the night, and in the morning was wandering, but tranquil. Mouth very sore; appetite good; pulse weak and low, 100. The bowels being in a confined state, he was ordered a dose of Castor oil.

April 28. From the 6th instant he had continued to mend progressively; the delirium occasionally returned till the seventh of the month, when it finally disappeared. He daily improved in his health, but particularly in the appearance of the ulcerations. It seemed that the last salivation had, at length, produced the desirable change in the habit, and had conquered the disease. From that time they had ceased to be painful, and very soon were found in a healthy condition.

By the latter end of June, the ulcer on the penis, and that in the groin, were both perfectly healed; and it is fair to conclude, that the disease was radically cured, because no topical applications whatever, except dry lint, had been used, from first to last.

This man, from his long indisposition, was allowed to remain in the hospital for some time after his complaints were cured.

---

*A Case, which proves that now and then, though perhaps rarely, the Medulla Oblongata will bear a considerable degree of pressure, without suffering any material derangement either in its structure or functions.*

[ With an Engraving. ]

A large robust woman, about forty years of age, had been for some years cook in a family. In the winter season she was employed in the kitchen, and while exerting herself to lift a weight, she fell down in a state of insensibility. She was supposed to have been in that condition for some time, when a person, accidentally coming in, found her; she was carried to bed, and continued in the same state for some hours, and then expired. She lay apparently asleep; respiration unattended with any stertorous sound; pulse small, beating about 100. She had not been altogether insensible, for when spoken to she had made several attempts to speak, in which she failed. She once had swallowed a little medicine offered her.

This woman had apparently been in good health for several years before; but latterly had always after dinner been been very apt to fall asleep. She was of a sedentary habit, and very fond of reading.

EXAMINATION.

Upon opening the head, the vessels upon the pia mater were found very turgid, and were very large and numerous. The brain more firm than usual.

The superior parietes of each hemisphere being pared, the right lateral ventricle was carefully opened, and within it was found a serous fluid tinged with blood. The plexus choroides was converted into a tuberculated structure.

The blood had passed through an opening from the left ventricle. A small coagulum was found arrested in its passage through the rupture in the septum lucidum. Within the left lateral ventricle a larger quantity of fluid, and a larger proportion of red blood, were found, than had been observed in the right. There was also a corresponding state of disease in the choroid plexus.

The fluid contents having been allowed to run off, the left corpus striatum appeared very tumid, greatly altered in colour, and of a livid hue; in two points it was lacerated, small coagula of blood were found entangled in these openings.

The striated substance of this part of the brain was now pared away, and beneath it, very near its surface, was found a very large mass of pure coagulated dark blood. The quantity of blood extravasated here was at least equal to four ounces, the whole of which had deposited itself in the medullary substance of the brain; extending of course into the anterior and posterior lobes of the cerebrum on the left side.

The internal carotid arteries were in some parts becoming opake, although they were not found ossified.

The present advanced state of physiological knowledge, has ascertained that the offices and uses of the medulla oblongata and medulla spinalis, are of so essential a nature, that while the superior parts of the brain will endure pressure, from any cause, to a considerable extent, the medulla oblongata, or medulla spinalis, cannot bear the least intrusion upon the space allotted to them, and that any extravasation of blood in these situations, even if only to the extent of a millet seed in bulk, proves fatal. In the progress of this examination, however, it was found that both the vertebral arteries, just above the point where they emerge from their canal in the bone, had undergone a considerable change, they were dilated so that each formed ed a little aneurysmal bag, which to the feel was harder and less elastic than the other parts of the canal.

Upon the medulla spinalis these tumours must have pressed, during the period of their growth, which, from the known history of aneurysmal tumors, might have been expected to have produced the symptoms from pressure for some months before. The woman never complained, or felt any deficiency in her state of health, till the accidental rupture of a blood vessel overwhelmed the brain, and terminated her existence.

These aneurysms in miniature were beautifully vascular upon their external surface.

*Mill Street, Hanover Square, June* 10, 1810.

( To be continued. )

## Figures and Tables

**Figure f1:**